# Improved therapeutic approach for spinal muscular atrophy via ubiquitination‐resistant survival motor neuron variant

**DOI:** 10.1002/jcsm.13486

**Published:** 2024-04-22

**Authors:** Joonwoo Rhee, Jong‐Seol Kang, Young‐Woo Jo, Kyusang Yoo, Ye Lynne Kim, Sang‐Hyeon Hann, Yea‐Eun Kim, Hyun Kim, Ji‐Hoon Kim, Young‐Yun Kong

**Affiliations:** ^1^ School of Biological Sciences Seoul National University Seoul South Korea; ^2^ Molecular Recognition Research Center Korea Institute of Science and Technology Seoul South Korea

**Keywords:** AAV, motor neuron, neuromuscular disease, neurotoxicity, SMA, SMN, spinal muscular atrophy, survival motor neuron, Zolgensma

## Abstract

**Background:**

Zolgensma is a gene‐replacement therapy that has led to a promising treatment for spinal muscular atrophy (SMA). However, clinical trials of Zolgensma have raised two major concerns: insufficient therapeutic effects and adverse events. In a recent clinical trial, 30% of patients failed to achieve motor milestones despite pre‐symptomatic treatment. In addition, more than 20% of patients showed hepatotoxicity due to excessive virus dosage, even after the administration of an immunosuppressant. Here, we aimed to test whether a ubiquitination‐resistant variant of survival motor neuron (SMN), SMN^K186R^, has improved therapeutic effects for SMA compared with wild‐type SMN (SMN^WT^).

**Methods:**

A severe SMA mouse model, SMA type 1.5 (*Smn* ^−/−^; *SMN2* ^+/+^; *SMN∆7* ^+/−^) mice, was used to compare the differences in therapeutic efficacy between AAV9‐SMN^WT^ and AAV9‐SMN^K186R^. All animals were injected within Postnatal Day (P) 1 through a facial vein or cerebral ventricle.

**Results:**

AAV9‐SMN^K186R^‐treated mice showed increased lifespan, body weight, motor neuron number, muscle weight and functional improvement in motor functions as compared with AAV9‐SMN^WT^‐treated mice. Lifespan increased by more than 10‐fold in AAV9‐SMN^K186R^‐treated mice (144.8 ± 26.11 days) as compared with AAV9‐SMN^WT^‐treated mice (26.8 ± 1.41 days). AAV9‐SMN^K186R^‐treated mice showed an ascending weight pattern, unlike AAV9‐SMN^WT^‐treated mice, which only gained weight until P20 up to 5 g on average. Several motor function tests showed the improved therapeutic efficacy of SMN^K186R^. In the negative geotaxis test, AAV9‐SMN^K186R^‐treated mice turned their bodies in an upward direction successfully, unlike AAV9‐SMN^WT^‐treated mice, which failed to turn upwards from around P23. Hind limb clasping phenotype was rarely observed in AAV9‐SMN^K186R^‐treated mice, unlike AAV9‐SMN^WT^‐treated mice that showed clasping phenotype for more than 20 out of 30 s. At this point, the number of motor neurons (1.5‐fold) and the size of myofibers (2.1‐fold) were significantly increased in AAV9‐SMN^K186R^‐treated mice compared with AAV9‐SMN^WT^‐treated mice without prominent neurotoxicity. AAV9‐SMN^K186R^ had fewer liver defects compared with AAV9‐SMN^WT^, as judged by increased proliferation of hepatocytes (*P* < 0.0001) and insulin‐like growth factor‐1 production (*P* < 0.0001). Especially, low‐dose AAV9‐SMN^K186R^ (nine‐fold) also reduced clasping time compared with SMN^WT^.

**Conclusions:**

SMN^K186R^ will provide improved therapeutic efficacy in patients with severe SMA with insufficient therapeutic efficacy. Low‐dose treatment of SMA patients with AAV9‐SMN^K186R^ can reduce the adverse events of Zolgensma. Collectively, SMN^K186R^ has value as a new treatment for SMA that improves treatment effectiveness and reduces adverse events simultaneously.

## Introduction

Spinal muscular atrophy (SMA) is a genetic disorder that affects the motor neurons controlling muscle movement in 1 per 6000–10 000 newborns.^1^ The survival motor neuron (SMN) protein is essential for the survival and function of motor neurons.^2^ The SMN1 gene produces most of the functional SMN protein, and mutations in the SMN1 gene result in a deficiency of SMN protein, which causes SMA. In particular, it is known that the onset of SMA is caused by low SMN levels, which cause motor neuron degeneration in the early postnatal period.^3^ In line with the need for a high expression level of SMN during the neonatal period in motor neurons, the neuromuscular system requires sufficient SMN protein for initial formation and early maturation.^4^, ^5^ Through an inducible transgenic approach for SMN induction in SMA mice, it has been confirmed that systemic induction of high levels of SMN at an early stage is essential not only for lifespan extension but also for improving the neuromuscular phenotype.^6^

One of the drugs used for early systemic restoration of SMN is Zolgensma, the adeno‐associated virus (AAV) that delivers an exogenous SMN1 gene to increase the SMN protein.^7^ The first clinical trial using Zolgensma (2 × 10^14^ viral genomes [VG]/kg) was conducted on post‐symptomatic SMA type 1 patients, and more than 60% of the patients failed to achieve developmental motor milestones.^8^ Recent clinical trials were performed on pre‐symptomatic SMA type 1 patients,^9^ selected through prenatal screening and newborn screening. Although pre‐symptomatic treatment showed better rescue effects than post‐symptomatic treatment,^8^, ^9^ motor skills were still insufficiently recovered in some patients.^9^, ^10^ This suggests that no matter how early the patient is treated, the therapeutic effect may be insufficient given the current efficacy of Zolgensma. Taken together, drug development for an improved therapeutic effect for SMA patients is still needed.

A major drawback of Zolgensma is the excessive virus dosage and the consequent adverse events of hepatotoxicity. A deleterious inflammatory response caused by the AAV leads to hepatocyte destruction, which results in liver failure.^11^, ^12^ According to the START trial study, albeit not fully achieving developmental motor milestones, high‐dose (2 × 10^14^ VG/kg) Zolgensma‐treated SMA patients showed improvements in motor functions. However, hepatotoxicity was observed in 25% of patients despite the administration of an immunosuppressant.^8^ In addition, in the phase III SPR1NT trial, hepatic adverse effects occurred in more than 20% of the Zolgensma‐treated pre‐symptomatic SMA patients, despite reducing the viral dose to 1.1 × 10^14^ VG/kg, the recommended dosage of Zolgensma.^9^ Especially low‐dose (6.7 × 10^13^ VG/kg) Zolgensma‐treated SMA patients showed no hepatotoxicity but showed no improvement in motor functions.^8^ For these reasons, SMA treatment using Zolgensma is currently being performed with a 1.1 × 10^14^ VG/kg viral dose, despite the accompanied hepatotoxicity. To minimize adverse events caused by excessive virus doses, Zolgensma treatment requires pretreatment with an immunosuppressant to reduce the inflammatory response to the virus.^13^ Nevertheless, hepatotoxicity has been reported in many SMA patients in several clinical trials to date.^8^, ^9^, ^10^, ^14^ Collectively, the results of these clinical trials underline the need to develop an AAV drug that not only achieves sufficient rescue effects but also reduces the hepatotoxicity caused by the immune response due to high‐dose virus treatment in SMA patients.

In this study, we improved the therapeutic effect of AAV on SMA through the delivery of a ubiquitination‐resistant SMN variant.^15^ In our previous report, we showed that the lysine at position 186 in SMN is critical for the post‐translational regulation and that the SMN^K186R^, which has a single nucleotide substitution of a lysine to arginine at position 186, is relatively more stable against ubiquitin‐proteasome system (UPS)‐dependent degradation compared with wild‐type SMN (SMN^WT^).^15^ Our study revealed that SMN^K186R^ extends lifespan, ensures the survival of motor neurons in the spinal cord and strengthens the overall neuromuscular system and motor function without neurotoxicity. This result suggests that SMN^K186R^ will show more remarkable effects in patients with severe SMA and have value as a new treatment that can reduce the adverse events of AAV at a low dose. Here, we provide a potential therapeutic strategy that is more effective and safe.

## Methods

### Animals

SMN2 (*SMN2* ^+/+^, *Smn* ^+/−^; stock 005024) and SMN2*delta7 (*SMN2* ^+/+^, *SMND7* ^+/+^, *Smn* ^+/−^; stock 005025) mice were obtained from the Jackson Laboratory. SMA type 1.5 (*SMN2* ^+/+^, *SMND7* ^+/−^, *Smn* ^−/−^) mice were generated by crossing SMN2 mice with SMN2*delta7 mice, and SMA type 1 (*SMN2* ^+/+^, *Smn* ^−/−^) mice and SMA type 2 (*SMN2* ^+/+^, *SMND7* ^+/+^, *Smn* ^−/−^) mice were produced by mating each type. Each mutant mouse was used to determine the difference in rescue effects from AAV injection. All mouse lines were housed under controlled conditions with specific pathogens and handled according to the guidelines of the Institutional Animal Care and Use Committee (IACUC) at Seoul National University.

### Newborn genotyping and adeno‐associated virus injection

The toes of newborn mice were cut in numerical order. PCR was performed using a genotyping primer set that can confirm the presence of mSmn. The amount of AAV suitable for each experiment was calculated and injected into the facial vein or cerebral ventricle of SMA mice within Postnatal Day (P) 1. AAV of 1.2 × 10^11^ VG or 4.0 × 10^10^ VG was prepared in 20 μL by mixing with phosphate‐buffered saline (PBS) for injection. Injection was performed using an insulin syringe with a 31‐G, 8‐mm‐long needle. The weights of the injected mice were checked daily thereafter.

### Righting reflex

A righting reflex test was performed starting at P5. The time taken from starting recording with the mouse lying down with its stomach facing upwards and turning its body over to stand upright was measured for 30 s. This process was repeated three times for each mouse, and the average time was calculated as one result.

### Negative geotaxis

A negative geotaxis test was performed on P20. The mouse was placed with its head facing downwards on a flat platform tilted at 50°, and the time it took to turn the body completely upwards was measured. Each trial was performed for 30 s, and if the body could not turn upwards at all, it was recorded as 30 s. The same process was repeated three times for each mouse.

### Hind limb clasping

A hind limb clasping test was performed after P20 or P40. After hanging the mouse by its tail, the mouse was suspended upside down in a vertical direction for 10 s and then recorded for 30 s. The time is measured by checking if the hind legs are not stretched outwards and if the legs are attached to the stomach or crossed. All motor function tests were conducted in a blinded manner to reduce the risk of researcher bias.

### Immunohistochemical (IHC) analysis

For cross‐sectional area (CSA) measurements, tibialis anterior (TA) muscles were embedded in O.C.T. Compound (Sakura Finetek) and snap‐frozen in liquid nitrogen (LN_2_), and each embedded muscle was sectioned at 7 μm using a cryostat. Slides were fixed with 4% paraformaldehyde (PFA) for 10 min, blocked with blocking buffer (5% goat and horse serum in PBS) and incubated with rat anti‐laminin (Abcam, ab11576, 1:1000) at 4°C overnight. The slides were incubated with Alexa Fluor 488‐conjugated anti‐rat IgG for 1 h at room temperature. Slides were mounted with VECTASHIELD and covered with coverslips. The muscle fibre size distribution for TA muscles in each treatment group was generated using the SMASH segmentation approach. Based on the muscle fibre size distribution, the average muscle fibre size was calculated for each mouse, and the same analysis was performed on all five mice in each treatment group.

### RNA extraction and qPCR

Liver tissues were homogenized to break down the cells and release their contents. The homogenate is then mixed with TRIzol Reagent (Life Technologies), and RNA is extracted according to the instruction manual. qRT‐PCR (QIAGEN) was performed with SYBR Green technology (SYBR Premix Ex Taq, QIAGEN) using *Igf‐1*, *Igfals*, *SMN* and a transduced AAV genome primer with *Gapdh* as a reference gene. Relative mRNA levels were determined using the 2^−ΔΔCt^ method.

### Statistical analysis

All statistical analyses were calculated by GraphPad Prism 9.2.0 (GraphPad Software). All variables were tested for normal distribution by using the Shapiro–Wilk test and QQ plot. Kaplan–Meier survival analysis combined with a log‐rank (Mantel–Cox) test was used to analyse animal lifespan. A one‐way analysis of variance (ANOVA) followed by the Bonferroni post hoc test was used to compare significant differences among multiple groups. A two‐way ANOVA followed by the Bonferroni post hoc test was used to compare multiple groups at multiple time points. A two‐tailed Student's *t*‐test was used to compare two groups of normally distributed data. All error bars represent the standard deviation of more than triplicate samples. The statistical significance was established at *P* < 0.05. Detailed experimental procedures are decribed in the supporting information.

## Results

### Improved therapeutic effects of ubiquitination‐resistant SMN^K186R^

**Figure 1:**
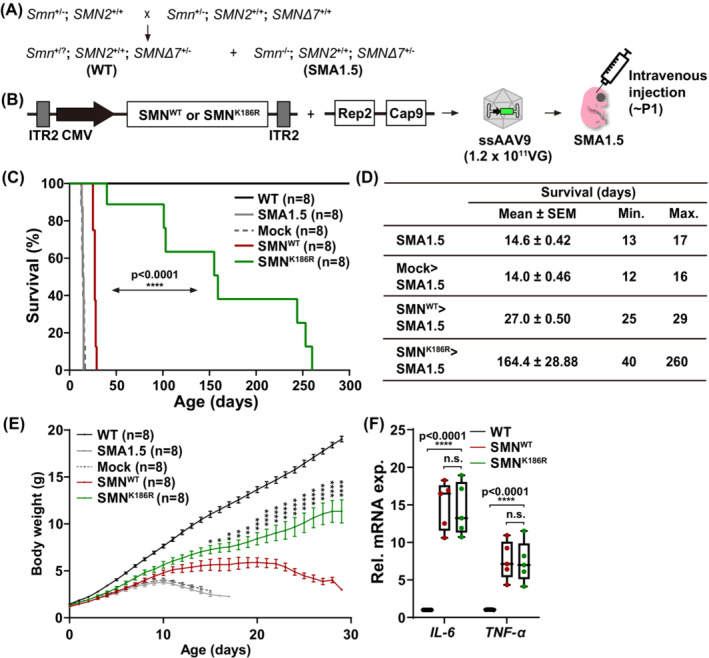
Lifespan extension and body weight increase in SMN^K186R^‐treated SMA type 1.5 mice. (A) Breeding scheme used to generate SMA type 1.5 mice (*Smn* ^−/−^; *SMN2* ^+/+^; *SMN∆7* ^+/−^). Heterozygous (HT) SMA type 1 (*Smn* ^+/−^; *SMN2* ^+/+^) mice were crossed with HT SMA type 2 (*Smn* ^+/−^; *SMN2* ^+/+^; *SMN∆7* ^+/+^) mice. SMA type 1.5 mutant mice were selected by genotyping primers capable of distinguishing the copy number of mSmn. Primers are listed in *Table* *S1*. (B) Experimental scheme of AAV injection. ssAAVs containing SMN^WT^ or SMN^K186R^ were generated by triple transfection and injected at 1.2 × 10^11^ VG through the facial vein within P1. (C) Kaplan–Meier survival curve of the SMA type 1.5 mice following treatment with ssAAV9‐Mock, ssAAV9‐SMN^WT^ or ssAAV9‐SMN^K186R^. Significant differences were observed between the two injected groups (log‐rank, *P* < 0.0001). *n* = 8 animals for each group. (D) Survival data obtained from injection with ssAAV9‐Mock, ssAAV9‐SMN^WT^ or ssAAV9‐SMN^K186R^ into SMA type 1.5 mice. (E) Body weight curves of SMA type 1.5 mice injected with ssAAV9‐Mock, ssAAV9‐SMN^WT^ or ssAAV9‐SMN^K186R^ compared with WT control and non‐injected SMA 1.5 mice. Each mouse was weighed once daily from birth. Data are mean ± SEM; two‐way ANOVA followed by the Bonferroni post hoc test; *P < 0.05, **P < 0.01, ***P < 0.001, ****P < 0.0001 for ssAAV9‐SMN^WT^ versus ssAAV9‐SMN^K186R^. The ANOVA revealed a statistically significant age‐by‐genotype (*P* < 0.001) interaction effect. (F) Relative mRNA expressions of inflammatory markers (*IL‐6* and *TNF‐α*) in the liver from ssAAV9‐SMN^WT^ or ssAAV9‐SMN^K186R^‐injected mice at P25. Data are mean ± SEM. ****P < 0.0001; by one‐way ANOVA followed by the Bonferroni post hoc test.

Ubiquitination assays of SMN from our previous study revealed that SMN^K186R^ is resistant to UPS‐dependent degradation.^15^ Based on previous research indicating that high SMN expression is required in the developmental stage,^4^, ^6^ we speculated that SMN^K186R^ might show better effectiveness in treating SMA mice compared with SMN^WT^. To date, many studies have reported that the degree of the rescue effect differs depending on the severity of the SMA mice.^7^, ^16^, ^17^ The most severe SMA type 1 (*Smn* ^−/−^; *SMN2* ^+/+^) mice have a short lifespan of ~7 days and show little treatment effect from Zolgensma.^17^ Mild SMA type 2 (*Smn* ^−/−^; *SMN2* ^+/+^; *SMN∆7* ^+/+^) mice have a lifespan of ~14 days and show impairment in motor functions, accompanied by neuromuscular junction (NMJ) defects. Zolgensma‐treated SMA type 2 mice showed a lifespan extension of more than 250 days, and several motor functions were improved.^7^ Therefore, we reasoned that both SMA type 1 and SMA type 2 mouse models are not suitable for testing the difference in therapeutic effects between SMN^WT^ and ubiquitination‐resistant SMN^K186R^, due to their precocious or latent phenotype emergence, respectively. Thus, we generated SMA type 1.5 (*Smn* ^−/−^; *SMN2* ^+/+^; *SMN∆7* ^+/−^) mice for injection (*Figure* *1* *A*). According to previous studies, SMA type 1.5 mice show NMJ defects earlier than SMA type 2 mice.^18^ Zolgensma uses self‐complementary AAV (scAAV), which has high transduction efficiency from bypassing rate‐limiting second‐strand synthesis.^19^ Based on the rescue results of SMA type 2 mice with Zolgensma,^7^ we also reasoned that the difference in therapeutic effects between SMN^WT^ and SMN^K186R^ on SMA type 1.5 mice can be masked when using scAAV. Thus, we generated single‐stranded AAV (ssAAV) containing SMN^WT^ or SMN^K186R^ to ensure slower transduction for better comparison (*Figure* *1* *B*). Based on previous studies showing that the cytomegalovirus (CMV) promoter has lower levels of transgene expression compared with the CMV immediate enhancer/β‐actin (CAG) promoter,^20^ we chose to use the ssAAV9‐containing CMV promoter.

To compare the therapeutic effects of SMN^WT^ and SMN^K186R^ on lifespan, we injected therapeutic doses (1.2 × 10^11^ VG)^21^, ^22^ of ssAAV9‐Mock, ssAAV9‐SMN^WT^ or ssAAV9‐SMN^K186R^ into the facial vein at P1. SMA type 1.5 mice and ssAAV9‐Mock‐treated mice had an average lifespan of 14 days and survived ~4 weeks by ssAAV9‐SMN^WT^ injection. To our surprise, ssAAV9‐SMN^K186R^‐treated mice had an average lifespan of 164 days, extending their lifespan by more than 140 days on average (*Figure* *1* *C,D*). The body weight of untreated SMA type 1.5 mice increased up to 2.5 g on average until P10 and then decreased until death at around P14. While mice injected with ssAAV9‐SMN^WT^ gained weight until P20 up to 5 g on average and then gradually decreased until death at around P28, mice injected with ssAAV9‐SMN^K186R^ continuously gained weight and size comparable with those of littermate wild‐type (WT) control, and this tendency continued even after 4 weeks (*Figure* *1* *E*). However, no significant differences in hepatic mRNA expression of interleukin‐6 (IL‐6) and tumour necrosis factor‐alpha (TNF‐α), markers of immune cell activation known to be upregulated due to AAV treatment,^23^ were observed between the two intravenously (i.v.) injected groups (*Figure* *1F*). These results show that ssAAV9‐SMN^K186R^ has greater therapeutic effects on lifespan and body weight than ssAAV9‐SMN^WT^ in SMA mice.

### Improved neuromuscular phenotypes by ssAAV9‐SMN^K186R^

**Figure 2:**
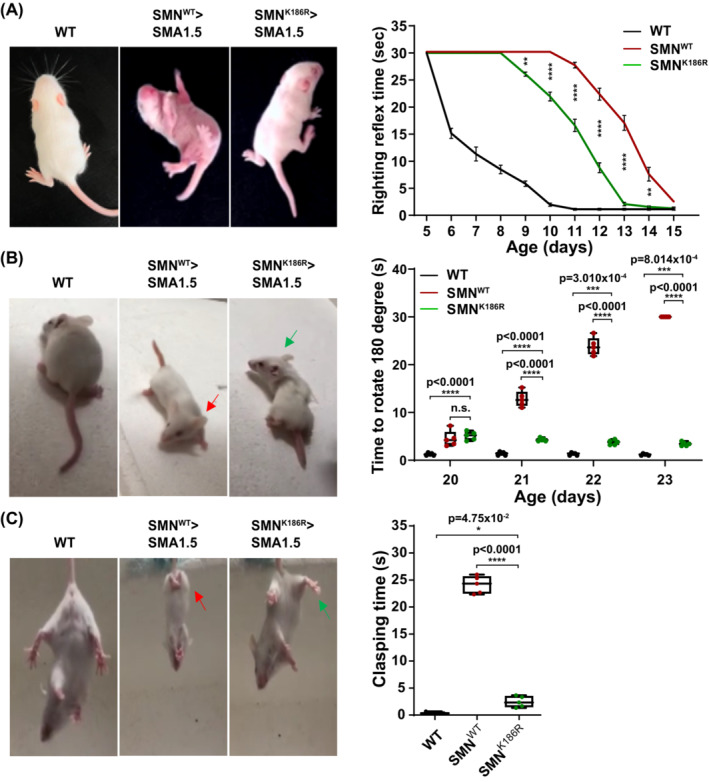
Enhanced motor function of SMN^K186R^‐treated SMA type 1.5 mice. (A) A righting reflex test was performed on P5, and representative images were obtained at P13. See also *Video* *S1*. Quantification time of returning to the prone position during the righting reflex test for 30 s. Quantitative analysis of the righting reflex time with *n* = 5 animals for each group. Data are mean ± SEM; two‐way ANOVA followed by the Bonferroni post hoc test; **P < 0.01, ****P < 0.0001 for ssAAV9‐SMN^WT^ versus ssAAV9‐SMN^K186R^. The ANOVA revealed a statistically significant age‐by‐genotype (*P* < 0.001) interaction effect. (B) A negative geotaxis test was performed on P20, and representative images were obtained at P22. See also *Video* *S2*. Quantification time of turning the body in an upward direction during the negative geotaxis test for 30 s. Quantitative analysis of the percentage of successful negative geotaxis and time to negative geotaxis with *n* = 5 animals for each group. Data are mean ± SEM; two‐way ANOVA followed by the Bonferroni post hoc test; *P < 0.05, **P < 0.01, ***P < 0.001. The ANOVA revealed a statistically significant age‐by‐genotype (*P* < 0.001) interaction effect. (C) A tail suspension test was performed at P23. See also *Video* *S3*. Quantification time of clasping hind limbs during the tail suspension test for 30 s. Quantitative analysis of the clasping time with *n* = 5 animals for each group. Data are mean ± SEM; one‐way ANOVA followed by the Bonferroni post hoc test; *P < 0.05, **P < 0.01, ***P < 0.001.

SMA mice show defective responses in the righting reflex, negative geotaxis and tail suspension tests.^24^ To examine these motor functions, we conducted a series of behavioural tests on ssAAV9‐SMN^WT^‐ or ssAAV9‐SMN^K186R^‐treated mice. In the righting reflex test, ssAAV9‐SMN^WT^‐treated mice showed successful righting reflexes from P11 on average, whereas ssAAV9‐SMN^K186R^‐treated mice showed successful righting reflexes from P9 on average (*Figure* *2* *A*). Also, there was a significant difference in righting time until P15, when the righting reflex time between the two injection groups became similar. In the negative geotaxis test, ssAAV9‐SMN^WT^‐treated mice gradually lost their ability to turn their bodies in an upward direction from P20 to P23, and all failed to turn upwards by around P23. On the other hand, latency to turn decreased in the ssAAV9‐SMN^K186R^‐treated mice with age, which became comparable with that of WT littermate control by P22 (*Figure* *2* *B*). In the tail suspension test, ssAAV9‐SMN^WT^‐treated mice displayed severe hind limb clasping, clasping for more than 20 out of 30 s. However, ssAAV9‐SMN^K186R^‐treated mice showed a clasping phenotype only for ~2 s, relatively similar to that of WT littermate control (*Figure* *2* *C*). Taken together, our observations show that ssAAV9‐SMN^K186R^ has better therapeutic effects on motor functions and coordination than ssAAV9‐SMN^WT^ in type 1.5 SMA mice.

**Figure 3:**
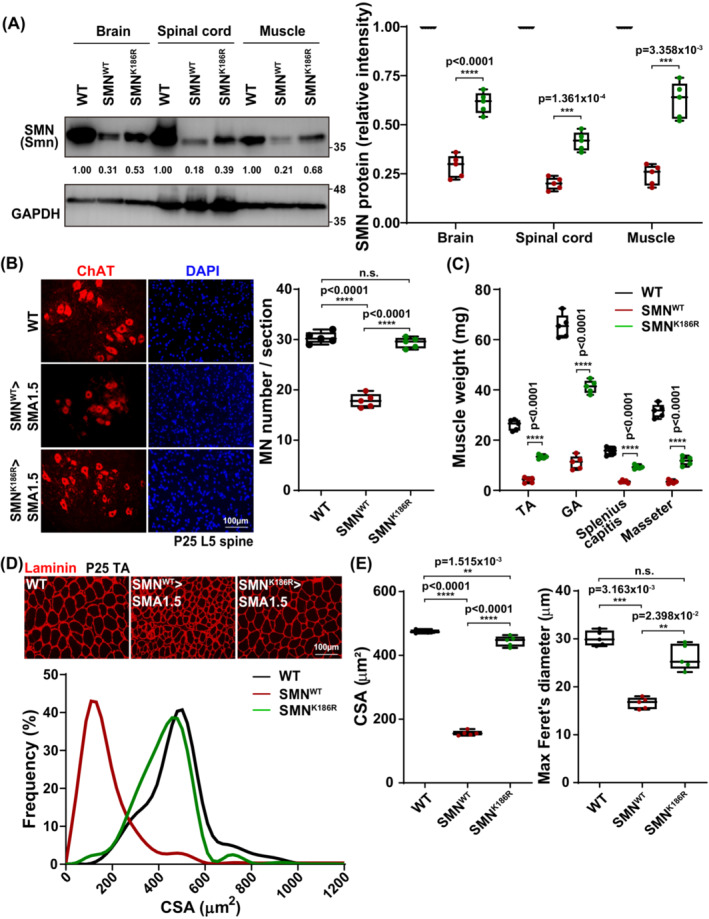
Improvement of the neuromuscular system of SMN^K186R^‐treated SMA type 1.5 mice. (A) Western blot analysis detecting SMN and GAPDH in brain, spinal cord and muscle samples with ssAAV9‐SMN^WT^‐ or ssAAV9‐SMN^K186R^‐injected SMA type 1.5 mice. Samples were immuno‐blotted with each indicated antibody. Data are mean ± SEM; one‐way ANOVA followed by the Bonferroni post hoc test; ****P < 0.0001. (B) Representative immunohistochemical (IHC) images for motor neurons (ChAT) and nuclei (DAPI) in ventral horn regions of the L5 spinal cord and quantification of MN number. Data are mean ± SEM; one‐way ANOVA followed by the Bonferroni post hoc test; **P < 0.01, ***P < 0.001 for ssAAV9‐SMN^WT^ versus ssAAV9‐SMN^K186R^. (C) Quantitative analysis of the muscle weight with *n* = 5 animals for each group. Data are mean ± SEM; one‐way ANOVA followed by the Bonferroni post hoc test; *P < 0.05, **P < 0.01 for ssAAV9‐SMN^WT^ versus ssAAV9‐SMN^K186R^. (D, E) Measurement of cross‐sectional area (CSA) distribution (D), mean CSA and maximum Feret's diameter (E) in TA muscles of ssAAV9‐SMN^WT^ or ssAAV9‐SMN^K186R^‐injected SMA type 1.5 mice at P25. Quantitative analysis of the mean CSA and maximum Feret's diameter with *n* = 5 animals for each group. Data are mean ± SEM. **P < 0.01, ***P < 0.001, ****P < 0.0001; by one‐way ANOVA followed by the Bonferroni post hoc test.

Symptomatic SMA mice show motor neuron loss and muscular defects caused by low SMN levels in the central nervous system (CNS) and muscles.^18^, ^25^ To investigate the rescue effects of ssAAV9‐SMN^K186R^ on the neuromuscular system, ssAAV9‐SMN^WT^‐ or ssAAV9‐SMN^K186R^‐treated mice were sacrificed at P25. As expected, relative SMN expression was higher in ssAAV9‐SMN^K186R^‐treated mice than in ssAAV9‐SMN^WT^‐treated mice in the brain, spinal cord and muscle (*Figure* *3* *A*). To further investigate the ubiquitination resistance of SMN^K186R^, we performed a cycloheximide (CHX) chase assay with MG132 (a proteasome inhibitor) and bafilomycin (an autophagy inhibitor) in SMA cells. As expected, relative SMN expression was higher in LV‐SMN^K186R^‐treated SMA cells than in LV‐SMN^WT^‐treated SMA cells in a ubiquitination‐dependent manner (*Figure* *S1*). In addition to this high SMN expression, the number of motor neurons, marked by motor neuron marker protein choline acetyltransferase (ChAT),^21^, ^26^, ^27^ was higher in ssAAV9‐SMN^K186R^‐treated mice than in ssAAV9‐SMN^WT^‐treated mice in the ventral horn of the L5 spinal cord (*Figure* *3* *B*). Also, the masses of limb or axial muscles were significantly increased in ssAAV9‐SMN^K186R^‐treated mice compared with those in ssAAV9‐SMN^WT^‐treated mice (*Figure* *3* *C*). When stained with laminin, a myofiber cell membrane marker,^28^ the CSA and maximum Feret's diameter were also increased in ssAAV9‐SMN^K186R^‐treated mice (*Figure* *3* *D,E*). Collectively, these data suggest that maintaining a relatively high level of SMN in motor neurons and muscles better improves the neuromuscular system, in terms of the number of motor neurons and the size of the muscle fibres.

### No prominent neurotoxicity in the intravenous delivery of ssAAV9‐SMN^K186R^

**Figure 4:**
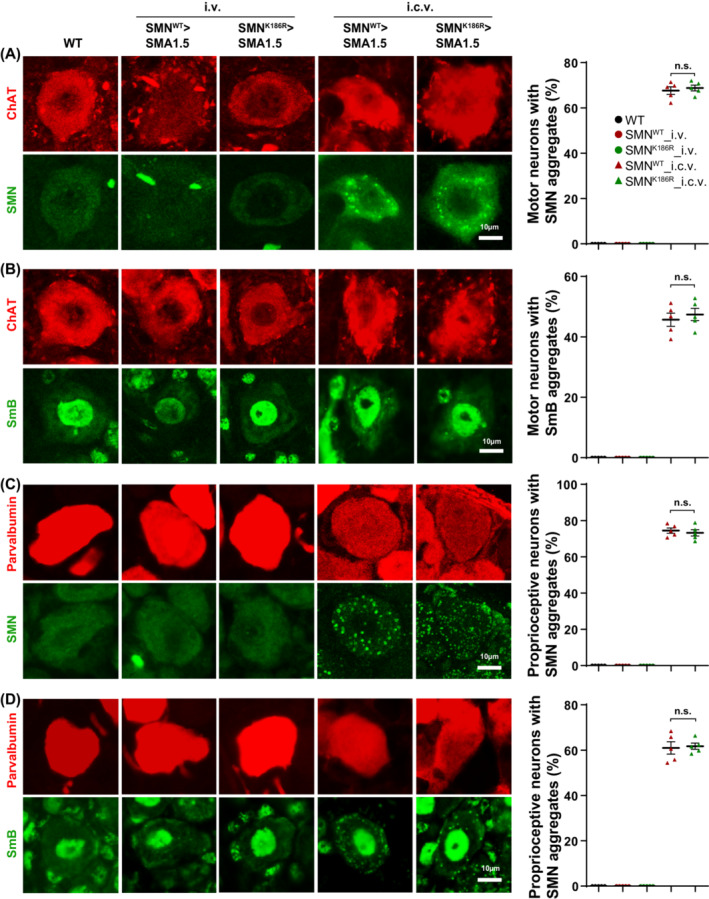
No prominent neurotoxicity in ssAAV9‐SMN^K186R^‐treated SMA type 1.5 mice. (A) Representative IHC images for motor neuron (ChAT) and SMN staining from mice injected with ssAAV9‐SMN^WT^ or ssAAV9‐SMN^K186R^ through the intravenous (i.v.) or intracerebroventricular (i.c.v.) route. Quantification of motor neurons with SMN aggregates at P25. Data are mean ± SEM; standard Student's *t*‐test; *P* < 0.05 considered significant. (B) Representative IHC images for ChAT and SmB staining from mice injected with ssAAV9‐SMN^WT^ or ssAAV9‐SMN^K186R^ through the i.v. or i.c.v. route. Quantification of motor neurons with SmB aggregates at P25. Data are mean ± SEM; standard Student's *t*‐test; *P* < 0.05 considered significant. (C) Representative IHC images for proprioceptive neuron (parvalbumin) and SMN staining from mice injected with ssAAV9‐SMN^WT^ or ssAAV9‐SMN^K186R^ through the i.v. or i.c.v. route. Quantification of proprioceptive neurons with SMN aggregates at P25. Data are mean ± SEM; standard Student's *t*‐test; *P* < 0.05 considered significant. (D) Representative IHC images for parvalbumin and SmB staining from mice injected with ssAAV9‐SMN^WT^ or ssAAV9‐SMN^K186R^ through the i.v. or i.c.v. route. Quantification of proprioceptive neurons with SmB aggregates at P25. Data are mean ± SEM; Student's *t*‐test; *P* < 0.05 considered significant.

A previous study reported that the delivery of scAAV9‐GUSB‐SMN^WT^ through intracerebroventricular (i.c.v.) injection sequestrates small nuclear ribonucleoproteins (SmB) by inducing cytoplasmic aggregation of SMN in motor neurons and dorsal root ganglion (DRG) accompanying hind limb clasping, which causes early signs of neurotoxicity.^29^ Based on our observations showing a different tendency for hind limb clasping defects in the two i.v. injected groups from P20 (*Figure* *2* *C*), we investigated whether early signs of neurotoxicity were different between the two groups. To test this possibility, we performed IHC analysis on the spinal cord and DRG of P25 SMA type 1.5 mice i.v. injected with ssAAV9‐SMN^WT^ or ssAAV9‐SMN^K186R^ at 1.2 × 10^11^ VG. Interestingly, neither abnormal cytoplasmic aggregation of SMN nor sequestration of SmB was observed in motor neurons in the two injection groups (*Figure* *4* *A,B*). Similarly, no signs of SMN aggregation or SmB sequestration were observed in the DRGs in both mice (*Figure* *4* *C,D*). This indicates that neurotoxicity caused by the accumulation of SMN in excess may not be sufficiently induced by i.v. delivery.

To test early signs of neurotoxicity in the i.c.v. injected condition, we i.c.v. injected 1.2 × 10^11^ VG of ssAAV9‐SMN^WT^ or ssAAV9‐SMN^K186R^ at P1 and analysed at P25. Interestingly, there was no difference in SMN aggregation and SmB sequestration in the motor neuron and DRG between the more stable SMN^K186R^‐injected mice and the SMN^WT^‐injected mice (*Figure* *4* *A–D*). Collectively, our data suggest that although AAV9‐delivered SMN^K186R^ has higher stability than SMN^WT^, it does not cause greater neurotoxicity in the CNS or show early signs of neurotoxicity when delivered i.v.

### Amelioration of liver defects in AAV9‐SMN^K186R^

**Figure 5:**
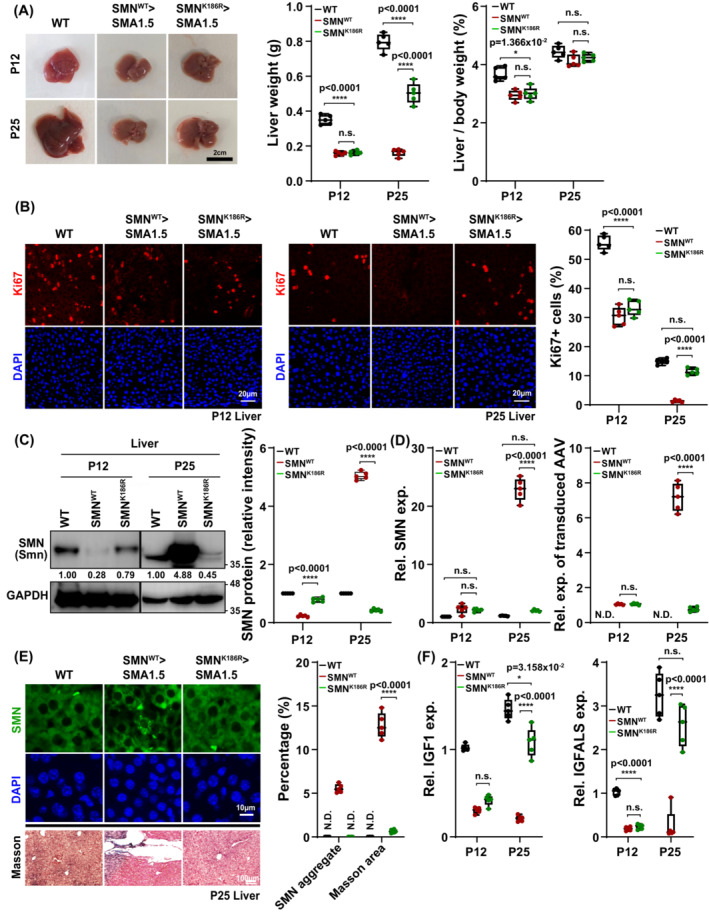
Improvement of liver defects in SMN^K186R^‐treated SMA type 1.5 mice. (A) Representative images of the liver in SMA type 1.5 mice following treatment with ssAAV9‐SMN^WT^ or ssAAV9‐SMN^K186R^ at P1. Quantitative analysis of the liver weight and the ratio of liver weight to body weight at P12 and P25 with *n* = 5 animals for each group. Data are mean ± SEM. **P < 0.01, ***P < 0.01, ****P < 0.0001; by one‐way ANOVA followed by the Bonferroni post hoc test. (B) Representative IHC images for Ki67 in hepatocytes at P12 or P25 and quantification of proliferating hepatocytes at P12 and P25. Data are mean ± SEM. *P < 0.05, ****P < 0.0001; by one‐way ANOVA followed by the Bonferroni post hoc test. (C) Western blot analysis detecting SMN and GAPDH in liver samples from ssAAV9‐SMN^WT^‐ or ssAAV9‐SMN^K186R^‐injected SMA type 1.5 mice. Samples were immuno‐blotted with each indicated antibody. Data are mean ± SEM; one‐way ANOVA followed by the Bonferroni post hoc test; ****P < 0.0001. (D) Relative mRNA expressions of SMN or transduced AAV genome in the liver from ssAAV9‐SMN^WT^‐ or ssAAV9‐SMN^K186R^‐injected SMA type 1.5 mice at P12 or P25. Data are mean ± SEM. ****P < 0.0001; by one‐way ANOVA followed by the Bonferroni post hoc test. (E) Representative IHC images for the accumulation of SMN and Masson's trichrome staining of liver from mice injected with ssAAV9‐SMN^WT^ or ssAAV9‐SMN^K186R^. Quantification of hepatocytes with SMN aggregates and liver fibrosis at P25. (F) Relative mRNA expressions of growth trait markers (*Igf‐1* and *Igfals*) in the liver from ssAAV9‐SMN^WT^‐ or ssAAV9‐SMN^K186R^‐injected mice at P12 or P25. Data are mean ± SEM. *P < 0.05, **P < 0.01, ***P < 0.001, ****P < 0.0001; by one‐way ANOVA followed by the Bonferroni post hoc test.

As ssAAV9‐SMN^K186R^‐treated mice showed greater weight gain compared with that of ssAAV9‐SMN^WT^‐treated mice after around P20 (*Figure* *1* *E*), we speculated that the two injection groups would have different liver weights, assuming that they would have the same liver‐to‐body weight ratios. Expectedly, the average liver weight of ssAAV9‐SMN^K186R^‐treated mice was significantly greater than that of ssAAV9‐SMN^WT^‐treated mice at P25, which became comparable when normalized to body weight (*Figure* *5* *A*). Based on the smaller absolute size of the liver in ssAAV9‐SMN^WT^‐treated mice, we investigated the differences in proliferation activity of hepatocytes between the two injection groups with the cellular marker for proliferation, Ki67.^30^, ^31^ The number of Ki67^+^ hepatocytes was comparable between ssAAV9‐SMN^WT^‐ and ssAAV9‐SMN^K186R^‐treated mice at P12 (*Figure* *5* *B*). Intriguingly, Ki67^+^ proliferating hepatocytes increased significantly in ssAAV9‐SMN^K186R^‐treated mice at P25, to levels nearly comparable with those of littermate controls, but were almost undetectable in ssAAV9‐SMN^WT^‐treated mice (*Figure* *5* *B*). This result showed that delivery of ssAAV9‐SMN^WT^ failed to achieve proper proliferation of hepatocytes in the SMA type 1.5 mice, compared with SMN^K186R^. As the episomal AAV genome is diluted out in highly proliferative cells, we speculated that SMN expression would be low in SMN^K186R^‐treated mice due to its higher proliferation. Indeed, the amount of SMN protein at P25 was less than that of P12 in ssAAV9‐SMN^K186R^‐treated mice. Surprisingly, we found that SMN was markedly accumulated in ssAAV9‐SMN^WT^‐treated mice (*Figure* *5* *C*). Consistent with the SMN protein accumulation, mRNA expression of SMN was also markedly increased in ssAAV9‐SMN^WT^‐treated mice but not in ssAAV9‐SMN^K186R^‐treated mice (*Figure* *5* *D*). Furthermore, qPCR analysis using primers that detect the transduced AAV genome also showed consistent results, where its levels were significantly higher in the ssAAV9‐SMN^WT^‐treated P25 mice (*Figure* *5* *D*). As AAV9‐mediated SMN overexpression is reported to cause motor function defects by the cytoplasmic aggregation of SMN in non‐dividing neurons, we speculated that the hepatocyte proliferation defect of ssAAV9‐SMN^WT^‐treated mice might have caused cytoplasmic aggregation of SMN. Intriguingly, cytoplasmic aggregation of SMN was formed only in ssAAV9‐SMN^WT^‐treated mice (*Figure* *5* *E*). To further investigate the difference in liver defects in two injected conditions, we performed Masson's trichrome staining and observed that the distribution of reactive fibrosis as a result of liver injury was observed only in AAV9‐SMN^WT^‐injected mice and not in AAV9‐SMN^K186R^‐injected mice (*Figure* *5* *E*). To test whether these pathological changes lead to functional defects in the liver, we performed RT‐qPCR for the genes *Igf‐1* and *Igfals*.^32^ Surprisingly, the expression levels of *Igf‐1* and *Igfals* were greatly increased in ssAAV9‐SMN^K186R^‐treated mice at P25, comparable with those of littermate controls, but were almost undetectable in ssAAV9‐SMN^WT^‐treated mice (*Figure* *5* *F*). Collectively, these data suggest that AAV9‐delivered SMN^K186R^ has better therapeutic effects in the liver than SMN^WT^ by promoting hepatocyte proliferation, increasing *Igf‐1* expression and decreasing abnormal cytoplasmic aggregation of SMN.

### Rescue of spinal muscular atrophy mice by low‐dose scAAV9‐SMN^K186R^

**Figure 6:**
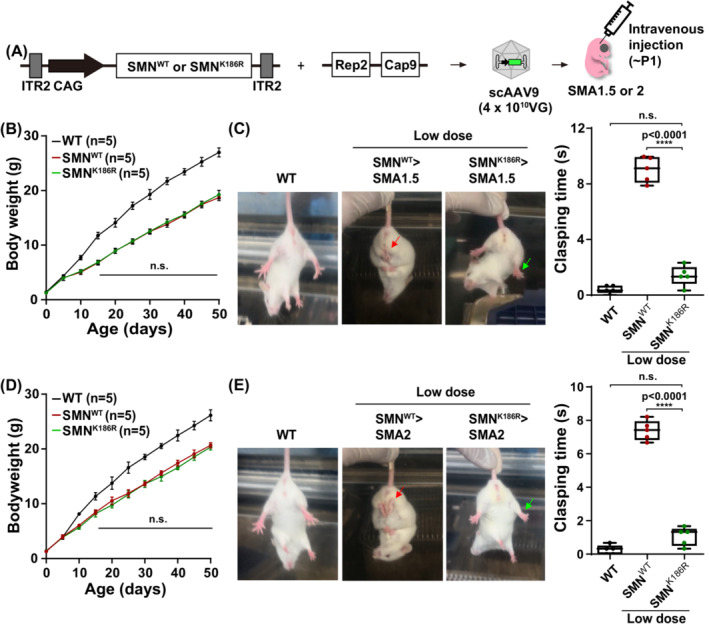
Motor function improvement in low‐dose SMN^K186R^‐treated SMA mice. (A) Experimental scheme of AAV injection. scAAVs containing SMN^WT^ or SMN^K186R^ were generated by triple transfection and injected at 4.0 × 10^10^ VG through the facial vein within P1. (B) Body weight curves of SMA type 1.5 mice injected with low‐dose scAAV9‐SMN^WT^ or scAAV9‐SMN^K186R^ compared with WT littermate control mice. Each mouse was weighed once daily from birth. Data are mean ± SEM; two‐way ANOVA followed by the Bonferroni post hoc test. The ANOVA did not reveal a statistically significant age‐by‐genotype (*P* = 0.9428) interaction effect. (C) A tail suspension test was performed after P40. See also *Video* *S4*. Quantification time of clasping hind limbs during the tail suspension test for 30 s. Quantitative analysis of the clasping time with *n* = 5 animals for each group. Data are mean ± SEM. ****P < 0.0001; by one‐way ANOVA followed by the Bonferroni post hoc test. (D) Body weight curves of SMA type 2 mice injected with low‐dose scAAV9‐SMN^WT^ or scAAV9‐SMN^K186R^ compared with WT littermate control mice. Each mouse was weighed once daily from birth. Data are mean ± SEM; two‐way ANOVA followed by the Bonferroni post hoc test. The ANOVA did not reveal a statistically significant age‐by‐genotype (*P* = 0.5807) interaction effect. (E) A tail suspension test was performed after P40. See also *Video* *S5*. Quantification time of clasping hind limbs during the tail suspension test for 30 s. Quantitative analysis of the clasping time with *n* = 5 animals for each group. Data are mean ± SEM. ****P < 0.0001; by one‐way ANOVA followed by the Bonferroni post hoc test.

Hepatotoxicity caused by an innate immune response to an excessive amount of AAV is still a major problem with Zolgensma.^33^ Therefore, research on lowering the viral dosage of AAV‐mediated therapy is still needed. As ssAAV9‐SMN^K186R^ delivery to SMA type 1.5 mice showed better rescue effects in terms of both survival and motor function than ssAAV9‐SMN^WT^, we speculated that low‐dose scAAV9‐SMN^K186R^ would also outperform the therapeutic effects of scAAV9‐SMN^WT^. Thus, we generated scAAV9‐SMN^WT^ and scAAV9‐SMN^K186R^ and injected them into neonatal SMA type 1.5 or 2 mice with 4 × 10^10^ VG i.v., which is one third of the clinical viral dose (*Figure* *6* *A*). Unlike the SMA type 1.5 mice injected with either ssAAV9‐SMN^WT^ or ssAAV9‐SMN^K186R^, no difference in weight gain was observed between low‐dose scAAV9‐SMN^WT^ and low‐dose scAAV9‐SMN^K186R^ even after P20 (*Figure* *6* *B*). Still, despite the increased weight of the mice, hind limb clasping was observed from around 6 weeks of age in scAAV9‐SMN^WT^‐treated mice. Intriguingly, mice injected with scAAV9‐SMN^K186R^ at the same low dose did not show any motor function defects (*Figure* *6* *C*). This hind limb clasping phenotype was also observed in SMA type 2 mice (*Figure* *6* *D,E*). Collectively, low‐dose injection of scAAV9‐SMN^K186R^ could be an innovative treatment strategy that can not only rescue motor function but also reduce adverse side effects from AAV dosage.

## Discussion

The current SMA treatment using Zolgensma has two major problems: insufficient therapeutic effects and hepatotoxicity caused by an excessive virus dose. Here, we showed that SMN^K186R^, ubiquitination‐resistant SMN, improves the therapeutic effect of AAV treatment and reduces virus dosage to alleviate adverse events on SMA compared with SMN^WT^. Our present study revealed that the systemic administration of AAV9‐SMN^K186R^ achieves a higher degree of SMN restoration than AAV9‐SMN^WT^ in both neural and non‐neural tissues. In line with the SMN restoration level, SMN^K186R^ extended longevity dramatically and improved the functional rescue effect of the neuromuscular system in the SMA mouse model, resulting in superior motor activity compared with SMN^WT^. Furthermore, SMN^K186R^ treatment had lower liver defects compared with SMN^WT^‐treated SMA mice. Thus, our results suggest the possibility of developing an alternative drug for Zolgensma by using SMN^K186R^ to achieve improved therapeutic efficacy and reduced adverse effects.

### Improved therapeutic efficacy of AAV9‐SMN^K186R^ on motor function

The long‐term follow‐up (LTFU) of the START study of Zolgensma monitored the patients for 5 years after treatment,^34^ focusing on motor function improvement and the maintenance of a healthy state in patients. All patients were alive during the period of LTFU. However, although additional improvements in motor milestones were observed in two patients, the other six patients only maintained the initial status of ‘sitting unassisted’ during the LTFU period. These results indicate that the present treatment with Zolgensma for SMA patients could be insufficient for long‐term rescue. Accordingly, these results suggest the need for an improvement in therapy effectiveness for SMA patients. In the present study, we showed durable and improved motor functions in an SMA mouse model after the treatment of AAV9‐SMN^K186R^ compared with that of AAV9‐SMN^WT^ (Zolgensma). AAV9‐SMN^K186R^‐treated mice showed remarkable improvement in several motor function tests and longevity. These results indicate that delivering SMN^K186R^ has a superior therapeutic effect than that of SMN^WT^. Thus, our study provides a new way to treat SMA patients more effectively by delivering SMN with higher stability.

### Ameliorated liver defects in AAV9‐SMN^K186R^ treatment

Immunoassays from previous research revealed that SMA patients have significantly lower levels of insulin‐like growth factor‐1 (IGF‐1) in their serum.^35^ As IGF‐1 is necessary for the general growth of individuals,^36^ restoration of the IGF‐1 level would be important for SMA patients whose height and weight have been reported to be lower than those of healthy individuals.^37^ Consistent with this, SMA mouse models show functional defects of the liver accompanying abnormal erythropoiesis and reduced IGF‐1,^38^ and the report that body weights were increased after systemic IGF‐1 restoration in SMA mice^39^ supports this notion. As the AAV genome is diluted out in actively dividing cells, the accumulation of SMN by AAV9‐SMN^WT^ delivery at P25 could have been caused by the impaired proliferation of hepatocytes. Unlike AAV9‐SMN^WT^, the gain of liver weight and body growth by AAV9‐SMN^K186R^ delivery might be due to the proper proliferation of hepatocytes and restored IGF‐1 production, although further studies are required to elucidate these unexpected observations. Nevertheless, these differences would not have been caused by the loaded AAV9 virus, as we used the same viral backbone and the same viral dosage. The only difference was a substitution from lysine to arginine in the 186th amino acid in SMN. Importantly, we could not observe any accumulation of SMN in the liver after AAV9‐SMN^WT^ delivery into WT or SMA type 2 mice (data not shown), suggesting that functional defects of the liver after AAV9‐SMN^WT^ delivery may be influenced by SMA severity for unknown reasons. Therefore, treatment of severe SMA patients using SMN^K186R^ can be an effective method to maximize functional improvement of the liver.

### Therapeutic efficacy of low‐dose AAV9‐SMN^K186R^

Controlling the balance between efficacy and safety is a significant issue in drug development.^40^ The first clinical trial of Zolgensma^8^ was conducted in high‐dose and low‐dose groups. In that study, none of the patients in the low‐dose group showed improved motor milestones compared with the high‐dose group, despite the low frequency of adverse events. In other words, it is necessary to consider the possibility that a low dose of Zolgensma could not sufficiently restore the level of SMN according to the developmental stage of SMA patients. Therefore, an improved AAV treatment with a better therapeutic effect at a lower viral dose is needed. In the present study, we observed motor function improvement after low‐dose treatment of AAV9‐SMN^K186R^, which was not observed in the low‐dose treatment of AAV9‐SMN^WT^. These results show the improvement of the rescue effect by delivering stable SMN^K186R^ using AAV9 at a low dose and suggest the low‐dose treatment of AAV‐SMN^K186R^ as a new therapeutic strategy that could achieve motor milestones successfully and reduce adverse events in SMA patients at the same time.

Recently, unexpected liver failure occurred in two SMA type 1 patients treated with Zolgensma, even after prednisolone pretreatment. Considering the improved therapeutic efficacy and less toxic characteristics of SMN^K186R^ in the liver, delivery of SMN^K186R^ with the AAV system to severe SMA patients may be a good alternative to prevent sudden death from liver failure.

In summary, Zolgensma, which was approved by the FDA in 2019, has shown therapeutic effects such as extending lifespan and improving exercise capacity for numerous SMA patients. However, side effects caused by excessive AAV and insufficient therapeutic effects are still pointed out as limitations in several clinical trials. Our research suggests that an improved therapeutic approach for SMA via ubiquitination‐resistant SMN, SMN^K186R^, will achieve better therapeutic effects in severe SMA newborn patients. Also, the enablement of low AAV dose treatment from the improved treatment effects of SMN^K186R^ provides strong foundations for clinical applications in SMA patients to reduce hepatotoxicity, a major side effect of Zolgensma.

## Conflict of interest statement

The authors declare that they have no conflicts of interest.

## Supporting information


**Figure S1.** Half‐life and ubiquitin/proteasome‐dependent proteolysis of SMN protein.

**Data S1.** Supporting Information.

**Movie S1.** **for Figure 2. SMA type 1.5 mice treated with 1.2 x 10** ^**11**^ **VG ssAAV9‐SMN** ^**WT**^ **or ssAAV9‐SMN** ^**K186R**^ **on P1 for righting reflex test.** WT littermate control and both‐treated mice were video‐recorded on P13 for comparing righting reflex. ssAAV9‐SMN^K186R^‐treated mice showed successful righting reflex compared to ssAAV9‐SMN^WT^‐treated mice.

**Movie S2.** **for Figure 2. SMA type 1.5 mice treated with 1.2 x 10** ^**11**^ **VG ssAAV9‐SMN** ^**WT**^ **or ssAAV9‐SMN** ^**K186R**^ **on P1 for negative geotaxis test.** WT littermate control and both‐treated mice were video‐recorded on P23 for comparing negative geotaxis. ssAAV9‐SMN^K186R^‐treated mice turned their body in the upward direction successfully compared to ssAAV9‐SMN^WT^‐treated mice.

**Movie S3.** **for Figure 2. SMA type 1.5 mice treated with 1.2 x 10** ^**11**^ **VG ssAAV9‐SMN** ^**WT**^ **or ssAAV9‐SMN** ^**K186R**^ **on P1 for tail suspension test.** WT littermate control and both‐treated mice were video‐recorded on P23 for comparing hind limb clasping. ssAAV9‐SMN^WT^‐treated mice showed severe clasping phenotype more than 20 out of 30 seconds.

**Movie S4.** **for Figure 6. SMA type 1.5 mice treated with 4.0 x 10** ^**10**^ **VG (low‐dose) scAAV9‐SMN** ^**WT**^ **or scAAV9‐SMN** ^**K186R**^ **on P1 for tail suspension test.** WT littermate control and both‐treated mice were video‐recorded on P40 for comparing hind limb clasping. scAAV9‐SMN^WT^‐treated mice showed clasping phenotype more than 8 out of 30 seconds.

**Movie S5.** **for Figure 6. SMA type 2 mice treated with 4.0 x 10** ^**10**^ **VG (low‐dose) scAAV9‐SMN** ^**WT**^ **or scAAV9‐SMN** ^**K186R**^ **on P1 for tail suspension test.** WT littermate control and both‐treated mice were video‐recorded on P40 for comparing hind limb clasping. scAAV9‐SMN^WT^‐treated mice showed clasping phenotype more than 6 out of 30 seconds.

**Table S1.** Supporting Information
