# Supplementary figures and images for: Improved therapeutic approach for spinal muscular atrophy via ubiquitination‐resistant survival motor neuron variant
Source: J Cachexia Sarcopenia Muscle. 2024 Apr 22;15(4):1404–17. doi: 10.1002/jcsm.13486 (PMC11294043; doi:10.1002/jcsm.13486)

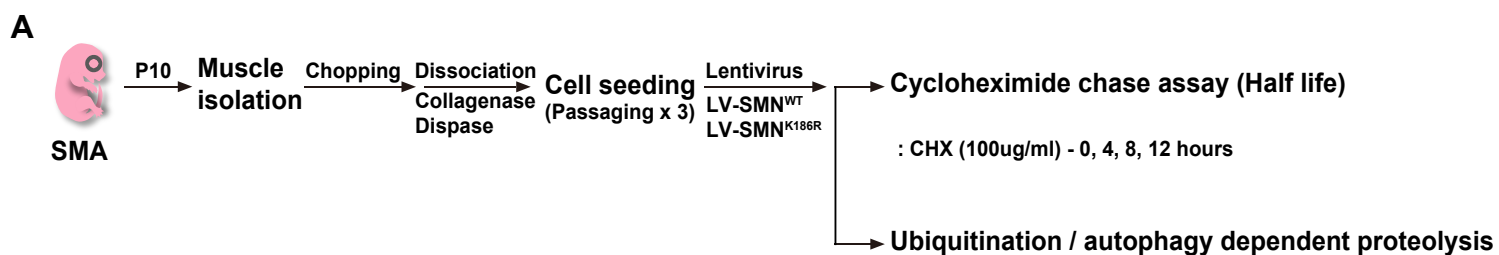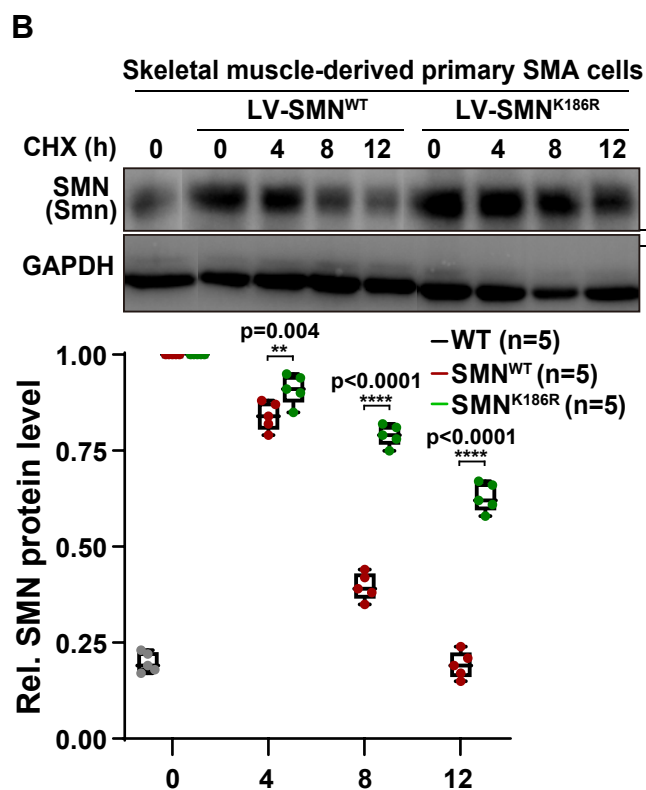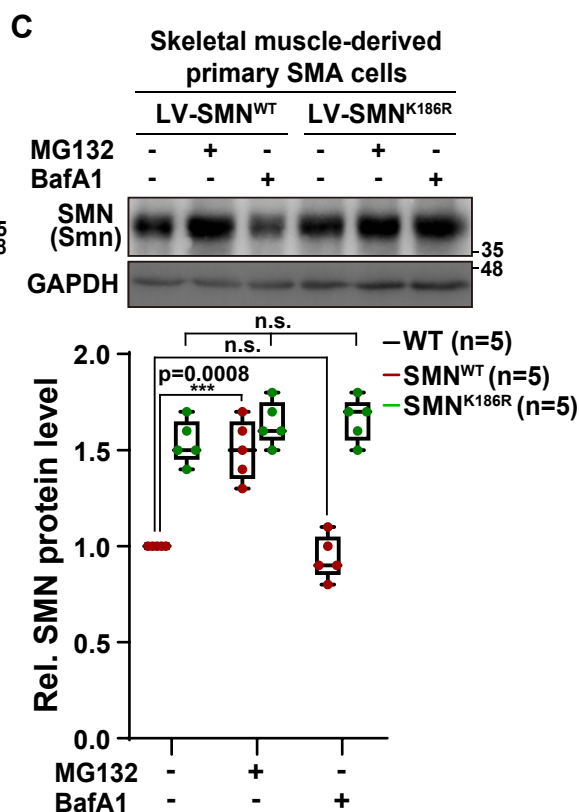

Supplement: Supplementary file 1 — Figure S1. Half‐life and ubiquitin/proteasome‐dependent proteolysis of SMN protein. [file JCSM-15-1404-s004.pdf]
