# Supplementary material for: Improved therapeutic approach for spinal muscular atrophy via ubiquitination‐resistant survival motor neuron variant
Source: J Cachexia Sarcopenia Muscle. 2024 Apr 22;15(4):1404–17. doi: 10.1002/jcsm.13486 (PMC11294043; doi:10.1002/jcsm.13486)
Supplement: Supplementary file 2 — Data S1. Supporting Information. [file JCSM-15-1404-s001.docx]

**Improved therapeutic approach for Spinal Muscular Atrophy via ubiquitination-resistant Survival Motor Neuron variant**

Joonwoo Rhee^1^, Jong-Seol Kang^1^, Young-Woo Jo^1^, Kyusang Yoo^1^, Ye Lynne Kim^1^, Sang-Hyeon Hann^1^, Yea-Eun Kim^1^, Hyun Kim^1^, Ji-Hoon Kim^2*^, and Young-Yun Kong^1*^

^1^School of Biological Sciences, Seoul National University, Seoul, South Korea; ^2^Molecular Recognition Research Center, Korea Institute of Science and Technology, Seoul, South Korea.

^*^ Correspondance - Ji-Hoon Kim and Young-Yun Kong

Lead contact - Young-Yun Kong
Email: [ykong@snu.ac.kr](mailto:ykong@snu.ac.kr); Tel: +82-2-880-2638; Address: 1 Gwanak-ro, Gwanak-gu, Seoul, 08826 South Korea.

Figure S1. Half-life and ubiquitin/proteasome-dependent proteolysis of SMN protein.

(A) Experimental scheme of cycloheximide (CHX) chase assay with MG132 and Bafilomycin in SMA cells. Enzymatically dissociated muscle cells were treated with CHX only (100 µg/ml), CHX + MG132 (50 µM), or CHX + Bafilomycin (400 nM) for the indicated times. (B and C) Western blot analysis detecting SMN and GAPDH in muscle samples with LV-SMN^WT^ or LV-SMN^K186R^-treated SMA muscle cells. Samples were immuno-blotted with each indicated antibody. Data are mean ± SEM; one-way ANOVA followed by the Bonferroni *post hoc* test; ****P* < 0.001.

**Supplementary materials**

**Methods**

*Cycloheximide (CHX) Chase Assay*

To obtain SMA cells, muscle tissues of SMA mice were dissociated enzymatically with DMEM containing collagenase II and dispase II for 1 hour at 37^o^C with mild agitation. After filtration through 40µm strainer, dissociated primary SMA cells were seeded in 60mm dish. Primary SMA cells transduced with lentiviral vectors containing His-tagged SMN^WT^ or SMN^K186R^ were subjected to immunoblot with His antibody. Transduced primary SMA cells were incubated with CHX only (100μg/ml, Sigma-Aldrich), CHX + MG-132 (50μM, proteasome inhibitor, for ubiquitin/proteasome-dependent proteolysis, Millipore-Sigma), or CHX + Bafilomycin (400nM, autophagy inhibitor, for autophagy-dependent proteolysis, Sigma-Aldrich) and harvested up to 24 hours at 4-hour intervals.

*AAV vector production*

*Transfection and purification.* pssAAV-Mock, pssAAV-CMV-SMN^WT^-2xflag, pssAAV-CMV-SMN^K186R^-2xflag (Cell Biolabs), pscAAV-CAG-SMN^WT^-2xflag or pscAAV-CAG-SMN^K186R^-2xflag vectors (Addgene) were co-transfected with pHelper plasmid (Takara) and Rep2/Cap9 plasmid (Addgene) into AAVproHEK293T cells (Takara) using polyethyleneimine (PEI; Polysciences). pssAAV-Mock, pssAAV-CMV-SMN^WT^-2xflag and pssAAV-CMV-SMN^K186R^-2xflag vectors were cloned using pssAAV-CMV-IRES GFP, and pscAAV-CAG-SMN^WT^-2xflag and pscAAV-CAG-SMN^K186R^-2xflag vectors were cloned using pscAAV-CAG-EGFP vectors as a backbone DNA. Serum starvated HEK293T cells were transfected with 14ug each at a pAAV:Red/Cap:pHelper ratio of 1:1:1. After 72 hours, cells were harvested with a cell scraper, centrifuged, and cell pellets obtained were lysed with lysis buffer (15 mL of 5 M NaCl, 25 mL of 1 M Tris HCl at pH 8.5, up to 500 mL with ultrapure water). The virus fraction obtained through 3 times-freeze-thaw processes was treated with benzonase (50U/ml; Sigma-Aldrich) to remove genetic material around AAV. After centrifuging the benzonase-treated virus fraction, the supernatant was filtered with a 0.45 uM pore filter and loaded onto an iodixanol (Sigma-Aldrich) gradient, and ultracentrifuged at 301,580g for 2 hours at 12°C. A needle was inserted into the 40% layer of the ultracentrifuged iodixanol gradient to obtain crude lysate and stored at 4°C.

*Desalting and concentration / Titration.* The crude lysate containing 5ml of 1x PBS-MK (dilution from 5x PBS-MK; 250 mL of 10x PBS, 2.5 mL of 1 M MgCl_2_, 6.25 mL of 1 M KCl, up to 500 mL with ultrapure water) was put into a pre-rinsed centrifugal tube and centrifuged at 4,000g until 1ml remained. After adding 13ml of 1x PBS-MK and repeating the same process three or more times, centrifugation was performed until the final volume remained at 200-300ul. Quantitative PCR (qPCR) analysis was performed for titration using this fraction and a standard curve samples. To remove any genetic material in the DNA fraction, 2ul of DNA fraction was mixed with 198ul of DNase 1 buffer (1 × 10^-2^ fraction) and treated with DNase 1 (Fisher scientific). It was maintained at 37°C for 30 minutes and DNase1 inactivation was performed at 95°C for 10 minutes. For serial dilution of DNA within AAV via AAV capsid removal, proteinase K (Sigma-Aldrich) was treated at 60°C for 50 minutes and inactivated at 95 degrees for 20 minutes. This fraction was serially diluted from 1 × 10^–3^ VG/μL to 1 × 10^–5^ VG/μL in triplicates. For standard curve, the AAV vector used for transfection was linearized with restriction enzyme and loaded on an 1.5% agarose gel to elusion the DNA. Based on the concentration and size of the eluted DNA, 10^9^ VG/μL plasmid stocks were produced, and then 10^7^ VG/μL to 10^1^ VG/μL stocks were prepared by serial dilution. The titer of AAV was determined by the threshold value of standard curve and DNA.

*Western blot*

Brain, spinal cord, muscle and liver were put into RIPA-buffer (50 mM TRIS HCl with pH 7.4; 150 mM NaCl, 1% Nonidet P-40; 0.5% sodium deoxycholate; 0.1% SDS; 2 mM MgCl2) with protease inhibitors and homogenized with homogenizer. The dissolved tissues were centrifuged at 13,000 rpm for 30 minutes and transferred the supernatant to new eppendorf tubes. To measure the protein concentration, Bradford reagent (Bio-Rad Laboratories) was diluted with RIPA and added to a 96 well plate in duplicate with each tissue supernatant. Based on each quantitative value quantified by SPECTROstar (BMG LABTECH), the same amount of protein was prepared in the same volume. Each normalized protein was run on a 10% polyacrylamide gel at 0.3 A for 1 hour and transferred to a PVDF membrane (Millipore Sigma) at 110 V for 1 hour. The membranes were blocked with 5% skim milk in PBS-T for 1 hour at room temperature and incubated with mouse anti-FLAG (1:2000 dilution), rabbit anti-GAPDH (1:5000 dilution) at 4^o^C overnight. After three time washes with PBS-T, the membranes were incubated with secondary antibody for 1 hour at room temperature and developed using Fusion solo chemiluminescence imaging system (Vilber).

*Masson’s trichrome staining*

Embedded liver slides were stained with pre-heated Bouin’s solution for 20 minutes. After washing steps, liver slides were put into Hematoxylin solution and BS-acid fucshin solution with several washing steps. After that, liver slides were put into Phosphomolybdic-phosphotungstic acid solution for 5 minutes and transferred to aniline blue solution without washing step. After washing step, liver slides were put into 1% acetic acid solution for 2 minutes and dehydrated with alcohol and xylene.

*Immunohistochemical (IHC) analysis*

For spinal cords (DRGs) and livers, embedded samples were sectioned at 14μm using a cryostat. Spinal cords (DRGs) were incubated in pre-heated EDTA antigen retrieval buffer (1mM EDTA, 0.05% Tween 20, pH 8.0) for 10 minutes, blocked with mouse IgG blocking reagent and blocking buffer (5% donkey serum) for 1 hour. Slides were incubated with goat anti-ChAT (Merck millipore, 1:100), rabbit anti-parvalbumin (Merck millipore, 1:500), mouse-anti SMN (BD Biosciences, 1:100), or mouse anti-SmB (Invitrogen, 1:100) at 4^o^C overnight. The next day, the slides were incubated with Alexa Fluor 594 donkey anti–goat IgG (1:400), Alexa Fluor 488 goat anti–mouse IgG (1:400), Alexa Fluor 594 goat anti–rabbit IgG (1:400) and Alexa Fluor 488 goat anti–mouse IgG (1:400) for 1 hour at room temperature. Livers were stained with Ki-67 (Abcam, 1:500) or mouse-anti SMN (BD Biosciences, 1:100) at 4^o^C overnight and incubated with Alexa Fluor 594 goat anti–rabbit IgG (1:400) for proliferation, and Alexa Fluor 488 goat anti–mouse IgG (1:400) for SMN aggregation. Slides were mounted with VECTASHIELD and covered with coverslips.

***CSA measurements***

**Measurements of myofiber size of TA muscle were conducted using a previously described protocol.[1, 2] Briefly, single channel fluorescence images of TA muscles were segmented with SMASH segmentations. Selected muscle section was automatically segmented via ‘initial segmentation’, determining fiber edges with watershed transformation. To obtain more accurately segmented images, initial-segmented images were revised by adjusting the pixel size (μm/pixel) in ‘initial segmentation parameter’ section. To remove any fibers below the minimum or above the maximum fiber area, images were revised by ‘Fiber filter’ section. Segmentation of analysis was applied with a segmentation filter (50 μm^2^ < CSA < 1500 μm^2^; eccentricity ≤0.95) on laminin-stained sections. Five animals per group were used for CSA measurements, and 500-1000 myofibers were measured for each mouse.**

**Supplementary references**

**S1. Smith LR, Barton ER. SMASH - semi-automatic muscle analysis using segmentation of histology: a MATLAB application. Skelet Muscle. 2014;4:21.**

**S2. Seo JY, Kang JS, Kim YL, Jo YW, Kim JH, Hann SH, et al. Maintenance of type 2 glycolytic myofibers with age by Mib1-Actn3 axis. Nat Commun. 2021;12:1294.**
