# Supplementary material for: Improved therapeutic approach for spinal muscular atrophy via ubiquitination‐resistant survival motor neuron variant
Source: J Cachexia Sarcopenia Muscle. 2024 Apr 22;15(4):1404–17. doi: 10.1002/jcsm.13486 (PMC11294043; doi:10.1002/jcsm.13486)
Supplement: Supplementary file 8 — Table S1. Supporting Information [file JCSM-15-1404-s008.pdf]

Table S1

Primer sequence used in this study	
Genes	Primer (5'-3')
Smn F	CTCCGGGATATTGCGATTG
Smn WT R	TTTCTTCTGGCTGTGCCTTT
Smn MT R	GGTAACGCCAGGGTTTTCC
SMN2 F	CTGACCTACCAGGGATGAGG
SMN2 WT R	CCCAGGTGGTTTATAGACTCAGA
SMN2 MT R	GGTCTGTTCTACAGCCACAGC
SMN delta7 F	TCCATTTCCTTCTGGACCAC
SMN delta7 R	ACCCATTCCACTTCCTTTTT
SMN qRT F	TGACATTTGGGATGATACAGCA
SMN qRT R	ACTGTTGTAAGGAAGCTGCA
Transduced AAV genome qRT F	CATGTTCATACCTCTTATCTTCCT
Transduced AAV genome qRT R	AAATGTCAGAATCATCGCTCT
IGF1 qRT F	TGAGGGGAGCCAATTACAAAGC
IGF1 qRT R	CCGGGCATGAAGACACACACAT
IGFALS qRT F	GTGAAAGCAAACAGAGCAG
IGFALS qRT R	CATTGACCACTGGAGACTG
